# Profiling and Isolation of Ten Rare Branched-Chain Alkylresorcinols in Quinoa

**DOI:** 10.3390/molecules28135220

**Published:** 2023-07-05

**Authors:** Tim Hammerschick, Walter Vetter

**Affiliations:** Institute of Food Chemistry, University of Hohenheim, D-70599 Stuttgart, Germany; tim_hammerschick@uni-hohenheim.de

**Keywords:** alkylresorcinol, centrifugal partition chromatography, countercurrent chromatography, quinoa, silver ion chromatography

## Abstract

Alkylresorcinols (∑ARs) are bioactive lipid compounds predominantly found in cereals. These amphiphilic compounds exist in a high structural diversity and can be divided into two main groups, i.e., 5-alkylresorcinols (ARs) and 2-methyl-5-alkylresorcinols (mARs). The pseudocereal quinoa has a very unique AR profile, consisting not only of straight-chain alkyl chains but also *iso*- and *anteiso*-branched isomers. Here, we describe a method for the isolation of such methyl-branched ARs and mARs from quinoa. The enrichment of the ∑AR fraction from the lipid extracts by centrifugal partition chromatography (CPC) was followed by ∑AR profiling using countercurrent chromatography (CCC) and GC/MS analysis of CCC fractions. A total of 112 ∑ARs could be detected, 63 of which had not been previously described in quinoa. Due to this high number of ∑ARs, the direct isolation of individual ARs was not possible using conventional CCC. Instead, the more powerful heart-cut mode was applied to enrich the target compounds. A final purification step—the separation of CCC-co-eluting mARs from ARs —was performed via silver ion chromatography. Altogether, ten rare branched-chain ∑ARs (five *iso*-branched mARs and five *anteiso*-branched ARs, including mAR19:0-*i* and AR20:0-*a*) were isolated with purities up to 98% in the double-digit mg range.

## 1. Introduction

Alkylresorcinols (ARs) is the summarizing term for a highly complex group of 1,3-dihydroxybenzene (resorcinol) derivatives within the family of lipids [1,2]. The presence of these amphiphilic biomolecules has been linked with diverse positive nutritional and bioactive properties, such as anticancerogenic effects. The structure of ARs inhibits the proliferation of human cancer cells, and in vitro studies have shown cytotoxicity against certain types of cancer [3,4,5,6]. In addition, ARs can influence many pathological and physiological immune-related processes and are involved in gene regulation and cell signaling [3,7]. ARs also have an antioxidant effect and inhibit, for example, the copper-induced oxidation of low-density protein [4,8,9,10]. Last but not least, ARs show antiparasitic and antimicrobial activity [4,9,11]. For example, the antifungal effect of ARs can protect the cereal grains from infestation against phytopathogens [12,13].

**Figure 1:**
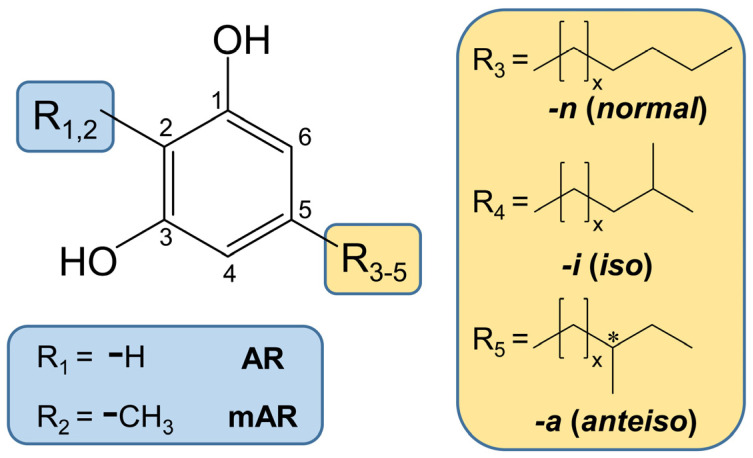
Chemical structure of 5-alkylresorcinols (ARs) and 2-methyl-5-alkylresorcinols ((m)ARs) with even- and odd-numbered alkyl chains in *normal*- (straight chained), *iso*-, and *anteiso*-configurations.

The reason for the high structural diversity of ARs lies in the variability of the alk(en)yl chain on C5 of the molecule. For example, for cereals, this substituent on C5 can vary in length (15–25 carbon atoms), as well as in the presence of double bonds (0–3), methyl branches (0–1), and keto (0–1) functions (Figure 1) [14,15,16]. The resulting long and complex chemical names have led to the introduction of short-hand abbreviations of the “ARn:m” type, where n denotes the length of alkyl side chain and m the number of double bonds (e.g., AR17:0 or AR17:2) [17]. Keto functions, such as in ARs in rye and wheat [18,19], can be indicated by the addition of “oxo” [17]. Methyl branches in the side chains may occur in *n*-2 (*iso* or *i*) and *n*-3 (*anteiso* or *a*) position [20]. The occurrence of methyl branches in the alkyl side chain can be referenced by adding “-*i*” or “-*a*” to the short-hand abbreviations (e.g., AR17:0-*i*) (Figure 1) [21].

The high structural variety of ARs is virtually doubled by compounds which additionally bear a methyl group on C2 (Figure 1) [20,22]. These 2-methyl alkylresorcinols (mARs) can be abbreviated by the same short type designation (“mARn:m”) [17].

Cereals such as rye, wheat, and spelt, which are particularly rich sources of ARs [14], were found to contain only traces, or no mARs [17,23]. However, the pseudocereal quinoa is comparably rich in mARs and also in methyl-branched ARs [20,24].

ARs and mARs (ΣARs) form a unique pattern in quinoa which could be useful for the authentication of products containing this pseudocereal [20,25]. However, neither standards of mAR nor methyl-branched ARs are currently commercially available, which also leads to the situation in which their uptake has hardly been studied and, accordingly, little knowledge exists with regard to their bioactivity.

For this reason, we aimed to profile the AR pattern of quinoa, followed by the isolation of several less common branched mARs and ARs by means of centrifugal partition chromatography (CPC) and countercurrent chromatography (CCC). CPC and CCC are related instrumental preparative chromatographic techniques which are based on the partitioning of the analytes in a biphasic solvent system. The separation takes place in a series of interconnected cells (CPC) or hollow tubes (CCC) filled with the biphasic solvent system, whereof one phase is kept stationary and the other one serves as the mobile phase. Separations are achieved when analytes differ in their partition coefficient (K_L/U_ value—quotient between the ratio of the analyte in the lower/upper phase) in the applied two immiscible phases [26,27,28,29]. Differently to rye [17], a direct profiling of quinoa by CCC was not possible due to its lower AR content paired with a higher lipid content, which necessitated an initial enrichment step. For this reason, we first applied CPC, which has a higher sample capacity than CCC (here, ~7–10 fold) at the expense of a lower resolution power [17,30,31]. Enriched ΣARs were fractionated using conventional CCC and analyzed via GC/MS. The isolation of individual ΣARs necessitated the application of the heart-cut mode for pre-purification. A final isolation and purification step was performed with silver ion chromatography (SIC).

## 2. Results and Discussion

Two bulk samples (3.5 or 3.0 kg sample) were extracted, one for AR profiling (sample-P, Section 2.2) and the other one for the isolation of rare branched-chain ΣARs (sample-I, Section 2.3). Before these CCC runs could be carried out, the ΣAR fraction was enriched from both bulk samples using CPC (Section 2.1).

### 2.1. Enrichment of Alkylresorcinols via Centrifugal Partition Chromatography (CPC)

Pooled cold extracts (CE, 46:54 *w*/*w*) of sample-P (~74.5 g from 3.5 kg quinoa seeds) and sample-I (~62.0 g from 3.0 kg quinoa seeds) corresponded with ~2.1% extracted lipids, which was more than four-fold the amount present in rye grains (~0.4–0.5% extract) [32,33]. These differences are in line with an almost four-fold higher fat content of quinoa seeds (~6.0%) compared to rye (~1.6%) [34]. In both cases, the extracts only yielded ~30% of the total lipids (~2.1% of 6% in quinoa and ~0.5% of 1.6% in rye). However, the extraction procedure adopted from Ross et al. aimed to gain ARs [20]. Since the total ΣAR content of quinoa seeds (~0.4 mg/g) and rye grains (~0.4–1.2 mg/g) [20] was comparable, the higher weight of the quinoa extract indicated a lower share of ARs, and this was linked with more efforts that were to be made for their enrichment and isolation. Under the assumption of an extraction yield of 80% ΣARs, quinoa extracts of sample-P (74.5 g) and sample-I (62.0 g) were expected to contain only ~1.1 g and ~1.0 g ARs, respectively, which is only 1.5% of the weight of the lipid extracts. These high lipid extract weights, along with low shares of ΣARs, were unsuited for the direct application of the present CCC system, which had a maximum sample load <1 g lipids [35]. Therefore, an initial enrichment step was implemented by means of CPC (sample capacity of the present CPC instrument: ~7 g [33]). In both cases, the injection of higher sample amounts was accompanied by the total loss of the stationary phase (also known as flooding).

The application of CPC in an ascending mode (nonpolar phase used as mobile phase) with the solvent system *n*-hexane/acetonitrile (1:1, *v*/*v*) led to a very fast elution of the predominant triacylglycerols (very nonpolar, K_L/U_ << 1). The first CPC run showed that the bulk of the sample matrix (but not the ΣARs) was eluted after 43 min (215 mL). After this point, ΣARs could therefore be obtained via elution extrusion (see experimental) due to the high K_L/U_ values of ~1.8–17 of AR25:0-AR15:0 [17]. Accordingly, a lot of time and solvent was wasted for the elution of the matrix. To overcome this drawback, a second sample aliquot was injected 7 min after the first one in the same CPC run. In this way, flooding could be prevented because the maximum sample capacity of the system was determined during the injection step [36].

Since the second sample was injected after 7 min, elution extrusion was started after 50 min (250 mL) instead of 43 min in the single injection run. Accordingly, >13 g sample could be fractionated in one CPC run with two injections, and solvent consumption and the total run time could roughly be halved in this operation mode. Several CPC runs were performed with aliquots until the matrix was separated from both bulk samples. Specifically, 93% (~69.6 g and ~57.8 g) of the weight of the extract could be removed and the remaining sample weight was reduced to 4.9 g sample-P and 4.2 g sample-I, respectively.

**Figure 2:**
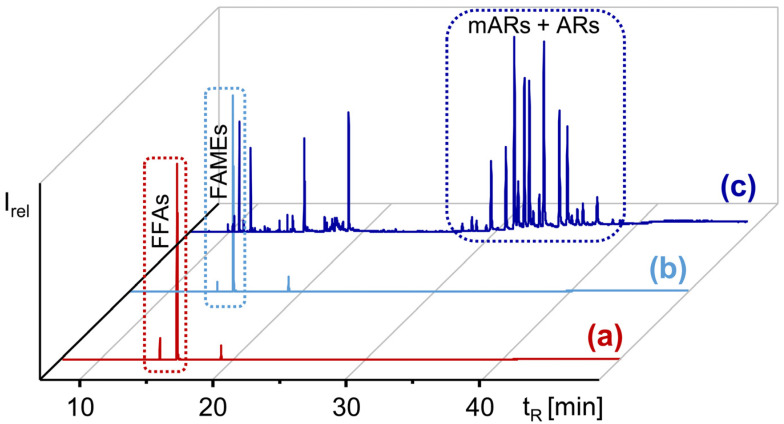
GC/MS chromatograms (full scan) of the silylated (**a**) ∑AR fraction enriched by CPC from quinoa extract, (**b**) methylated ∑AR fraction enriched by CPC from quinoa extract, and (**c**) the ∑AR fraction after a second CPC separation step with the methylated ∑AR extract shown in (**b**).

The GC/MS analysis of silylated aliquots indicated that ΣARs were detectable by extracting the base peaks (*m*/*z* 282 of mARs and *m*/*z* 268 of ARs, see experimental) from the GC/MS full scan chromatogram. However, the most relevant peaks originated from free fatty acids (FFAs), specifically 16:0, 18:1Δ9, 18:2Δ9,12, 18:3Δ9,12,15, and 18:0 (Figure 2), i.e., the major fatty acids in quinoa [34,37]. Since the FFAs were known to co-elute with ARs in CCC over a wide range [17], their removal was deemed indispensable for further ΣAR analysis.

This was carried out by the conversion of the FFAs into their corresponding less polar methyl esters (FAMEs), which then could be removed by a second CPC separation step from the ΣARs. Accordingly, the CPC fractionation of methylated pools of sample-P and sample-I (see experimental, Figure 2) provided ~950 mg sample-P and ~817 mg sample-I (~1.3% of the initial lipid extracts). ΣARs dominated, with ~80% of the samples (Figure 2), and were accompanied by unknown impurities. The further purification and isolation of ΣARs was performed with CCC (Section 2.2 and Section 2.3), whose chromatographic efficiency is superior to CPC [33,38].

### 2.2. Profiling of ΣARs in Quinoa with Countercurrent Chromatographic Fractionation Followed by GC/MS Analysis

**Figure 3:**
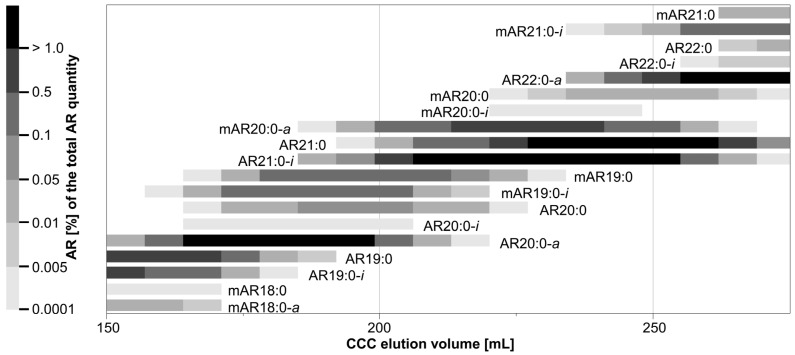
CCC elution volumes (excerpt) of saturated 5-alkylresorcinols (AR) and 2-methyl-5-alkylresorcinols (mAR) with their percentage share of the total AR distribution in the conventional CCC 2_H→T_ separation in head-to-tail mode with the solvent system *n*-hexane/ethyl acetate/methanol/water (9:1:9:1, *v*/*v*/*v*/*v*). Other ARs and mARs are not displayed.

An aliquot (~68%) of sample-P (after CPC) was CCC-separated in head-to-tail mode with the biphasic solvent system *n*-hexane/ethyl acetate/methanol/water (9:1:9:1, *v*/*v*/*v*/*v*) [17]. After a pre-run of 80 mL, 80 CCC fractions (CCC in head-to-tail mode) and 60 CCC fractions (CCC in tail-to-head mode) of 7 mL each were collected, respectively. Aliquots were taken from each fraction, silylated, analyzed using GC/MS, and CCC elution profiles were created (Section 3.4.3). Depending on the amount, very abundant ΣARs could occur in up to 26 CCC fractions, while minor ΣARs were only partly detected in one CCC fraction. In head-to-tail mode (lower, more polar phase used as mobile phase), saturated ΣARs with a short alkyl-chain on C5 eluted first (here, mAR15:0-*i*, AR15:0-*i*), while long-chained ones eluted last (here, mAR25:0-*i* and AR26:0-*a*) (SI Figure S2). By contrast, mARs co-eluted with their isomeric ARs (e.g., mAR19:0 and AR20:0) (Figure 3, SI Figures S1 and S2) [17], but *iso*- and *anteiso*-branched ARs eluted slight faster than the corresponding straight chained *n*-isomer (Figure 3, SI Figures S1 and S2). In most cases, both *iso-* and *anteiso*-isomers were detected in sample-P, but with varied relevance. Specifically, *anteiso*-isomers were predominant in the case of ARs and mARs with an even-numbered alkyl chain, while *iso*-isomers were prevailing in the case of odd-numbered ARs and mARs.

The present data produced strong evidence that in the biosynthesis of ARs and mARs, the alkyl residues originated from fatty acids. For the biosynthesis of ΣARs in the *anteiso*-configuration, the even-numbered ΣARs-*a* could arise from odd-numbered *anteiso*-fatty acids, which have the amino acid isoleucine as a primer [39,40]. In turn, the odd-numbered ΣARs-*i* would be formed from even-numbered fatty acids in *iso*-configuration, having the amino acid valine as a primer [39,40]. Unlike ΣARs-*i*, ΣARs-*a* have a stereo-center at the antepenultimate carbon. Unfortunately, it was not possible to determine the enantiomeric composition of the isolates. Based on the plausible hypothesis that the amino acid isoleucine is involved in the synthesis, it is rather likely that ΣARs-*a* are non-racemic an show a distinct predominance of the (*S*)-enantiomer, similar to *anteiso*-fatty acids [41,42,43].

Altogether, 112 ΣARs were detected in sample-P, which was more than twice the number previously reported in quinoa, i.e., 49 ΣARs by Ross et al. [20], Navarro del Hierro et al. [24], and Hammerschick and Vetter [21] without the use of CCC (Table 1). Most of the previously unreported ΣARs in quinoa were the less abundant monounsaturated (n = 14), diunsaturated (n = 6), triunsaturated (n = 1; AR21:3), and (monoun)saturated keto-ARs and -mARs (Table 1).

Due to the highly complex ΣAR profile with many structural variants, individual ΣARs could not be isolated in this way (Figure 3, SI Figure S2). Specifically, individual CCC fractions featured at least four ΣARs, and the purity of the most interesting but rare mARs and methyl-branched ARs was typically <60%.

In a further experiment, the remaining share of sample-P (~32%) was fractionated in tail-to-head mode, in which the upper less-polar phase was used as mobile phase (SI Figure S1). As a consequence, the elution order of mARs and ARs was reversed. However, the isomers that co-eluted above also co-eluted in tail-to-head mode (e.g., mAR19:0 and AR20:0 (SI Figures S1 and S2)) [17]. Vice versa, *iso*- and *anteiso*-branched ΣARs eluted slightly slower than the corresponding straight-chained *n*-isomers.

Hence, the isolation of rare ΣARs such as mAR19:0-*i* and AR20:0-*a*, which were (minor) contributors to their corresponding CCC elution range (Figure 3), required the application of an improved CCC method. For instance, mAR19:0-*i* and AR20:0-*a* were interesting target compounds whose CCC elution ranges were fully overlapping. Accordingly, this problem was virtually impossible to solve by means of CCC. However, a recent investigation indicated that mARs and ARs can be fully separated from each other by silver ion chromatography (SIC) [21].

However, CCC fractions with mAR19:0-*i* and AR20:0-*a* additionally contained seven further only partly co-eluting ΣARs, namely mAR18:0, mAR18:0-*a*, mAR20:0-*a*, AR19:0, AR19:0-*i*, AR21:0, and AR21:0-*i* (Figure 3). However, these only partly overlapping ARs can be removed by means of CCC operated in the more powerful heart-cut mode [44]. In addition, the same scenario (full/partial overlap) also existed for other chain lengths, and solving one problem will also solve other problems.

Last but not least, the results of this section indicated that the head-to-tail mode (K values of AR15:0-AR25:0: ~0.3–2.6) was better suited for ∑AR profiling than the tail-to-head mode (K values of AR25:0-AR15:0: ~0.4–3.3), mainly due to the shorter run times (~75 min) and lower solvent consumption (~150 mL). Hence, sample-I was subsequently used for the isolation of rare ΣARs by CCC in heart-cut mode (Section 2.3).

### 2.3. Isolation of Rare ∑ARs by Countercurrent Chromatography in Heart-Cut Mode (HC-CCC)—Method Description and Execution

#### 2.3.1. Method Description

CCC operated in heart-cut mode (HC-CCC) is particularly successful because the partial transfer of (major) compounds from the first (1st) dimension generates a focusing effect in the second (2nd) dimension [44]. The present CCC system features four coils in two bobbins which can be driven independently, and HC-CCC can be implemented by installing switching valves and wiring [45]. Previous applications in our group used bobbin 1 (coil 1 + 2) in the 1st dimension and bobbin 2 (coil 3 + 4) in the 2nd dimension [44,45,46]. In the present research, coils 2 + 3 (present in different bobbins, 236 mL) were used in the 1st dimension and coil 1 + 4 (also present in different bobbins, 235 mL) in the 2nd dimension. This change had the advantage that the elution volumes determined in Section 2.2 (where coil 2 + 3 was also used) could be directly adopted to determine the ranges of HCs (in the case of the same retention of the stationary phase (S_f_ value)). The elution volumes of the HCs were calculated as a function of the S_f_ value. This mode of proceeding allowed run-to-run variations and the loss of stationary phase after sample injection to be compensated, which explained the small discrepancies between the intended and the performed HC (Table 2). This, in turn, was of particular importance because HCs could not be monitored by UV signals due to the many UV-active ΣARs in the sample.

In this step, the goal was to enrich six pairs of ΣARs in six different HCs (Table 2). Each pair consisted of one methyl-branched AR and one methyl-branched mAR isomer (e.g., AR20:0-*a* with mAR19:0-*i*), both of which accompanied the other, only partly co-eluting ARs and mARs (Section 2.2).

As already mentioned, the length of the HC from the first to the 2nd dimension was selected from the CCC elution profiles created in Section 2.2, in such a way that provided as much as possible of the analytes and as little as possible of the co-eluting compounds. Afterwards, the transferred elution range with the focused analytes was further chromatographed in the 2nd dimension, and fractionated with the same goal. The collected sub-fractions were screened by GC/MS, and suitable ones were pooled for further purification via SIC (Section 2.4). In either case, the goal was to achieve purities of >95%. Exemplarily, the procedure will be explained for one of the pairs in Section 2.3.1.

**Figure 4:**
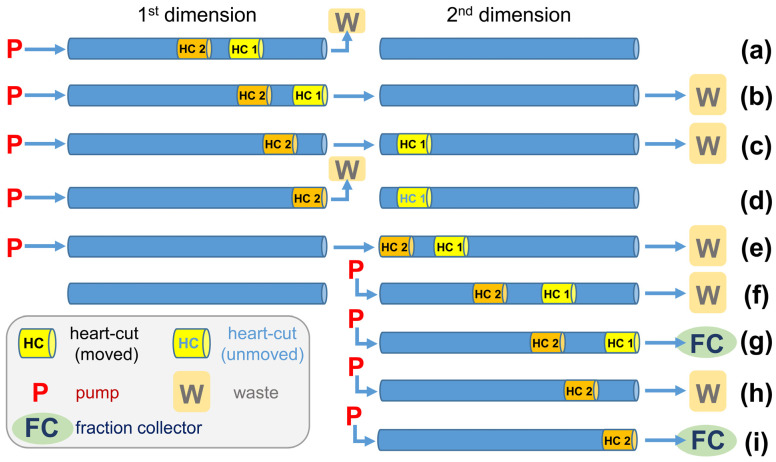
Schematic illustration of a CCC separation in heart-cut mode with two heart-cuts (HC) separated simultaneously in the 2nd dimension. (**a**) Pre-separation of the target compounds of HC 1 + 2 in the 1st separation. (**b**,**c**) Transfer of HC 1 to the 2nd dimension. (**d**) Only elution of the 1st dimension (HC 2 to the end of the 1st dimension). (**e**) Transfer of HC 2 to the 2nd dimension and the simultaneous separation of HC 1. (**f**) Simultaneous separation of HC 1 and HC 2 in the 2nd dimension. Simultaneous separation of HC 2 and fractionation of HC 1 (**g**). Separation of HC 2 in the 2nd dimension (**h**) with subsequent fractionation (**i**).

Increasing the elution volumes in the 1st dimension, and subsequently also in the 2nd dimension, was linked with a higher demand for solvents. To overcome this drawback, HC were separated simultaneously in the 2nd dimension (Figure 4). Specifically, the 1st dimension was moved until HC 1 had reached the end of the coil, while the flow was sent to waste (Figure 4a). Then, the flow was sent to the 2nd dimension until HC 1 was completely transferred to the 2nd dimension (Figure 4b,c). Afterwards, for a brief period, only the 1st dimension was moved until HC 2 had reached the end of the coil (Figure 4d). This was followed by the transfer of HC 2 to the 2nd dimension (Figure 4e). From this point on, the mobile phase only served the 2nd dimension (Figure 4f–i), and eventually HC 1 and HC 2 were directed individually to the fraction collector (Figure 4g–i) or could be fractionated together without step h) (Figure 4h).

**Figure 5:**
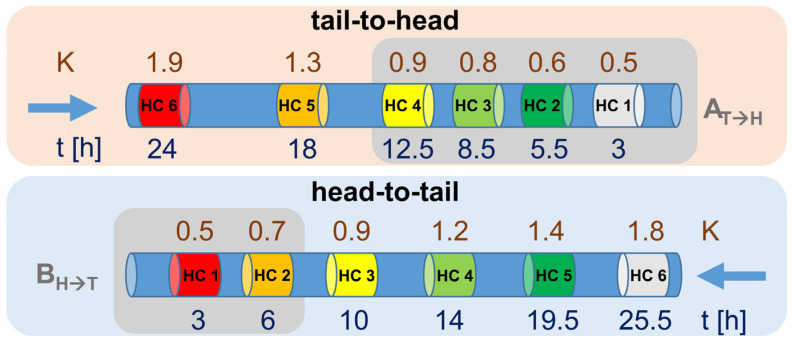
Schematic representation of the six relevant heart-cuts (HC) for the twelve target compounds using CCC in tail-to-head and head-to-tail modes. K values (K) reflect the approximate starting point of the beginning of the transfer area of the HC. Time (t) is the approximate estimated time after complete fractionation of each HC from the 2nd dimension. The grey areas are the actual HCs performed in HC runs A and B.

As a further point, for the wide K_L/U_ range of the ΣARs of ~0.3–2.6, the fractionation of the entire sample in one HC-CCC run would have been very time-demanding (estimated at >24 h). Instead, two runs were performed, with one in tail-to-head mode (HC-CCC A_T→H_ for the range AR25:0-AR21:0; *K* range ~0.40–1.25) and the second one in head-to-tail mode (HC-CCC B_H→T_ for the range AR15:0-AR20:0; *K* range ~0.30–0.85, Figure 5). By splitting the HC-CCC separations into HC-CCC A_T→H_ with four HC and HC-CCC B_H→T_ with two HC for the more polar target analytes, it was possible to reduce the estimated total time by ~1/3 (Figure 5). This also had the advantage that the separations could now be performed on two different days. An unattended HC separation was not possible, as the individual HC steps had to be set manually.

#### 2.3.2. Description of the Fractionation with the Example of AR20:0-*a* with mAR19:0-*i*

**Figure 6:**
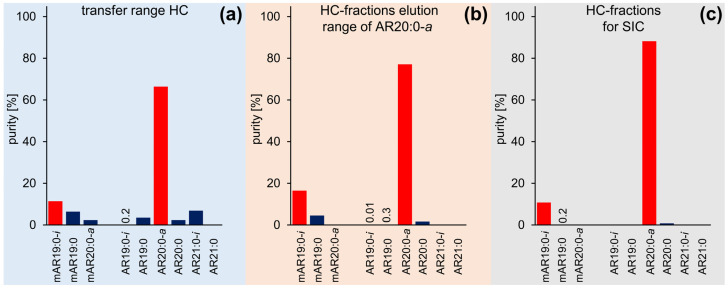
Purities of the target compounds mAR19:0-*i* and AR20:0-*a* and further co-eluting ∑ARs during CCC fractionation (**a**) in the transfer range of the heart-cut (172–205 mL), (**b**) in the elution range of AR20:0-*a* of the heart-cut fractionation (225–290 mL), and (**c**) in the heart-cut fractions pooled for silver ion chromatography (239.5–269.5 mL).

AR20:0-*a* with mAR19:0-*i* (HC 2 of HC-CCC B_H→T_) were fully co-eluting and their entire elution range (150–220 mL) additionally featured shares of nine additional ∑ARs. The reduction of the HC range to 172–205 mL (SI Figure S4) allowed the full removal of two ∑ARs while the share of the seven remaining and (partially) co-eluting ∑ARs was ~25%, compared to ~10% mAR19:0-*i* and ~65% AR20:0-*a* (Figure 6a). The subsequent separation and collection of the fraction 225–290 mL in the 2nd dimension (SI Figure S4) increased the purities of mAR19:0-*i* and AR20:0-*a* to ~16% and ~77% (with only minute contributions of mAR19:0, AR19:0-*i*, AR19:0, AR20:0, ∑~7%) (Figure 6b). Still, this good result could be further improved by the selection of 239.5–269.5 mL (SI Figure S4), which reduced the number of co-eluting compounds to mAR19:0 and AR20:0 (Figure 6c) and featured ~11% mAR19:0-*i* and ~88% AR20:0-*a*; this sample would later be subjected to SIC (Section 2.4). This strategy of HC 2 of HC-CCC B_H→T_ (mAR19:0-*i* and AR20:0-*a*) was also applied to the other HCs.

#### 2.3.3. HC Run A with Four Heart-Cuts (HC 1-HC 4)

HC-CCC A_T→H_ run (four HCs) successively transferred and completed the following four HCs with the listed elution ranges, i.e., (i) mAR23:0-*i* and AR24:0-*a* (HC 1, 132–151 mL), (ii) mAR22:0-*a* and AR23:0-*i* (HC 2, 151–173 mL), (iii) mAR21:0-*i* and AR22:0-*a* (HC 3, 188–212 mL), and (iv) mAR20:0-*a* and AR21:0-*i* (HC 4, 212–289 mL). In HC-CCC, only the active dimension was served with mobile phase. That means that once the desired transfer from the 1st dimension to the 2nd dimension was completed, the mobile phase was only sent to the 2nd dimension, while the flow was stopped in the 1st dimension (Figure 4f–i) [45]. Once the separation (of the first HC) was completed in the 2nd dimension, the mobile phase was sent back to the 1st dimension and the next HC could be performed, a.s.o. (Table 2, SI Figure S3a–d). However, during the transfer of HC 1, all subsequent pairs assigned to subsequent HC were also moved, so these volumes had to be added (Figure 4b,c). For this reason, HC 2 was started immediately after HC 1 was finished in the 2nd dimension, and HC 4 was also started immediately after HC 3 was completed (Figure 4c,e; step Figure 4d omitted). Only in the case of HC 3 was a delay of 15 mL introduced in which no sample was transferred to the 2nd dimension (Figure 4d).

In theory, the elution range in the 2nd dimension should be about 2 × √2 (double length plus wide widening effect in chromatography). However, the fractionation covered a larger range in order to elute and collect the additionally separated partly co-eluting compounds. This was particularly important for compounds with a longer elution range because the 2nd dimension had to be free of compounds when the next HC started. In addition, it was also possible to obtain further fractions in these marginal areas with purified ΣARs, some of which were pooled and used for SIC. Once the fourth HC was completed in the 2nd dimension, the remaining compounds in the 1st dimension were gained by elution extrusion and subjected to the second HC-CCC run (HC-CCC B_H→T_).

#### 2.3.4. HC run B with Two Heart-Cuts (HC 1 and HC 2)

The second HC-CCC B_H→T_ run included two HCs (Table 2). The elution volume of HC 1 (134–156 mL) covered ~75% mAR18:0-*a* and the first 55% of AR19:0-*i*. HC 2 (172–204.5 mL) transferred ~80% of mAR19:0-*i* and AR20:0-*a* to the 2nd dimension (Table 2, SI Figure S4).

Generally speaking, the main problem in all HC-separations was the co-elution of the methyl-branched target compounds with their unbranched isomers (e.g., mAR19:0-*i* with mAR19:0 and AR20:0-*a* with AR20:0). Despite slightly differing elution profiles and the improvement achieved by the HC mode, a full separation could not be achieved. Accordingly, a compromise had to be made between yield and purity of the target analyte, and also with regard to the subsequent SIC fractionation.

Note that during the transfer of HC 2, the target analytes of HC 1 were separated in the 2nd dimension at the same time. In this way, HC 2 and HC 3 in the HC-CCC A_T→H_ run as well as HC 1 and HC 2 in the HC-CCC B_H→T_ run were separated simultaneously after the transfer of the elution ranges of the HCs (SI Figures S3C and S4). The combined separations saved working load, time (~150 min and ~125 min), and solvents (~300 mL and ~250 mL). While it is possible to transfer the entire sample by subsequent HCs from the 1st to the 2nd dimension, this approach does not provide the focusing effect that is obtained by the partial removal of (abundant) matrix compounds [44]. To use this effect, there must be a gap between two HCs, which was excellently fulfilled in the present case.

In conclusion, CCC in HC mode was a powerful tool for the further purification of individual ∑ARs from a very complex AR matrix (112 ∑ARs in total). Although the HC mode also did not allow the direct isolation of individual mARs or ARs, as shown above, the number of co-eluting ∑ARs was reduced by specifically selected HCs and elution regions with purified mixtures of mARs, and ARs could now be used for complete purification by SIC. The conventional CCC, on the other hand, could only be used as a useful tool to study the ∑AR profile in quinoa compared to previous studies, where after the ∑AR profiling [17] isolation was also possible [32]. The reason for this is the choice of matrix and thus the ∑AR profile, including the number and structural variants of the ∑ARs and their elution behavior in the CCC. While the ∑AR profile from rye after hydrogenation consisted mainly of only six odd-numbered saturated ARs (AR15:0–AR25:0), which were sufficiently separated from each other by conventional CCC and can thus be obtained in high purity [32], the ∑AR profile from quinoa consisted of even and odd-numbered ARs as well as mARs, which were also present in different configurations (straight chain and branched in *iso* and *anteiso*-configuration). This large number of ∑ARs eluted close together, as shown above, so the use of CPC-CCC coupling as described by Hammerschick and Vetter [33] would not be of any use here either. The only option with CCC was to use the powerful HC mode.

### 2.4. Final Purification of (Methyl)Alkylresorcinols by Silver Ion Chromatography (SIC)

**Figure 7:**
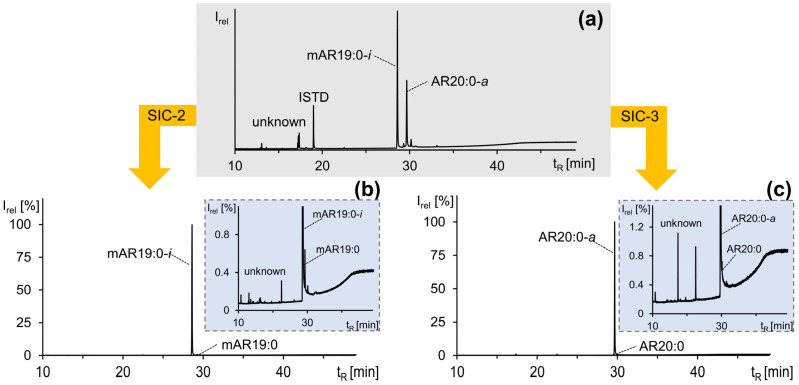
GC/MS chromatograms of (**a**) a fraction of the pooled heart-cut CCC fraction used for SIC, (**b**) isolated mAR19:0-*i* (>98% purity) of SIC fraction 2, and (**c**) isolated AR20:0-*a* (>94% purity) of SIC fraction 3. The minor impurities are shown in the enlarged excerpts in (**b**,**c**).

Using the solvent mixture *n*-hexane/ethyl acetate (92:8, *v*/*v*), it was recently found that SIC enabled the separation of saturated mARs (fraction SIC-2) from saturated ARs (fraction SIC-3, *n*-hexane/ethyl acetate (80:20, *v*/*v*)) [21]. Due to the presence of impurities, the fractionation scheme of Hammerschick and Vetter [21] was modified by adding a second 50 mL SIC-2 fraction (SIC-2.2) and by the subdivision of fractions SIC-2.1 and fraction SIC-3 into five smaller fractions of 10 mL each to increase the purities of the mARs as well as ARs. However, impurities also often eluted directly in SIC-1, so these fractions and fraction SIC-2.2 (partial elutions of further impurities that would otherwise elute in fraction SIC-3, reducing the purities of the ARs) were necessary for the higher purity of the target analytes. In addition, the amount and variance of the impurities differed, since they were also distributed by CCC separation according to their partition coefficients; there were never the same impurities and amounts that had to be separated by SIC to purify the target ΣARs. Unfortunately, SIC could not separate the different configurations of the alkyl side chain from each other, and therefore it was of crucial importance to achieve the highest possible purity by pooling selected CCC fractions after the HCs (Table 3). For example, in the SIC of pooled CCC fractions of HC 1 + 2 of the HC-CCC B_H→T_ run, the main unknown impurities were separated (Figure 7), but in fraction SIC-2.1 low amounts of mAR19:0 still reduced the purity of the target analyte mAR19:0-*i* (Figure 7b), while AR20:0 reduced the purity of AR20:0-*a* in fraction SIC-3 (Figure 7c). However, it was possible to isolate and purify ten different ΣARs in *iso*- and *anteiso*-configurations to >98% purity and up to double-digit mg levels (Table 3) from the mixture of 112 ΣARs and other compounds.

Recorded ^1^H NMR spectra of mARs confirmed the additional methyl group located on the resorcinol ring by the signal at 1.99 ppm and the absence of the signal typical for ARs at ~6.07 ppm caused by the proton at C2 of the resorcinol backbone (SI Table S1, Figure S6), which was in agreement with the literature data [20,47]. Likewise, the configuration of the branched side chains could be confirmed by the signal at 0.88 ppm (doublet) in six-fold relative intensity (integral I = 6) caused by two terminal methyl groups (*iso*-configuration) of mAR19:0-*i*, mAR21:0-*i*, and AR21:0-*i* (SI Table S1, Figure S6). In contrast, ^1^H NMR measurements of isolated mAR18:0-*a*, (m)AR20:0-*a*, (m)AR22:0-*a*, and (m)AR24:0-*a* gave chemical shifts at 0.87 ppm (triplet) and at 0.86 (doublet), caused by the terminal methyl group and the branched methyl group with I = 3, respectively, verifying the *anteiso*-configuration (SI Table S1, Figure S6) [20]. GC/MS spectra (SI Figure S5) in combination with ^1^H NMR spectra (SI Figure S6) of the isolated compounds consolidated the assignment of the mARs and ARs in different configurations by means of the logarithmic retention time plots [21].

Neither standard substances for saturated ARs in *iso*- and *anteiso*-configuration nor any standards of mARs regardless of the configuration were commercially available. Thus, the isolated ΣARs are of valuable importance and could now be used as reference standards, especially as specific biomarkers for quinoa and its uptake (food and human plasma) by the unique ∑AR profile [20,25], or also for bioactivity studies, for the investigation of their properties compared to the studied saturated ARs with straight-chain side chains, or generally the influence of the additional methyl group on the 1,3-dihydroxybenzene backbone of mARs.

## 3. Materials and Methods

### 3.1. Quinoa Sample and Chemicals

Several 500 g packages of whole, light beige Bolivian organic quinoa seeds were purchased as sample materials from a retail shop in Stuttgart, Germany. Acetonitrile was obtained from Bernd Kraft (Duisburg, Germany). Suppliers and grade of the remaining solvents, chemicals, and reagents were as specified by Hammerschick and Vetter [21].

### 3.2. Extraction of Alkylresorcinols (ARs) from Quinoa

**Figure 8:**
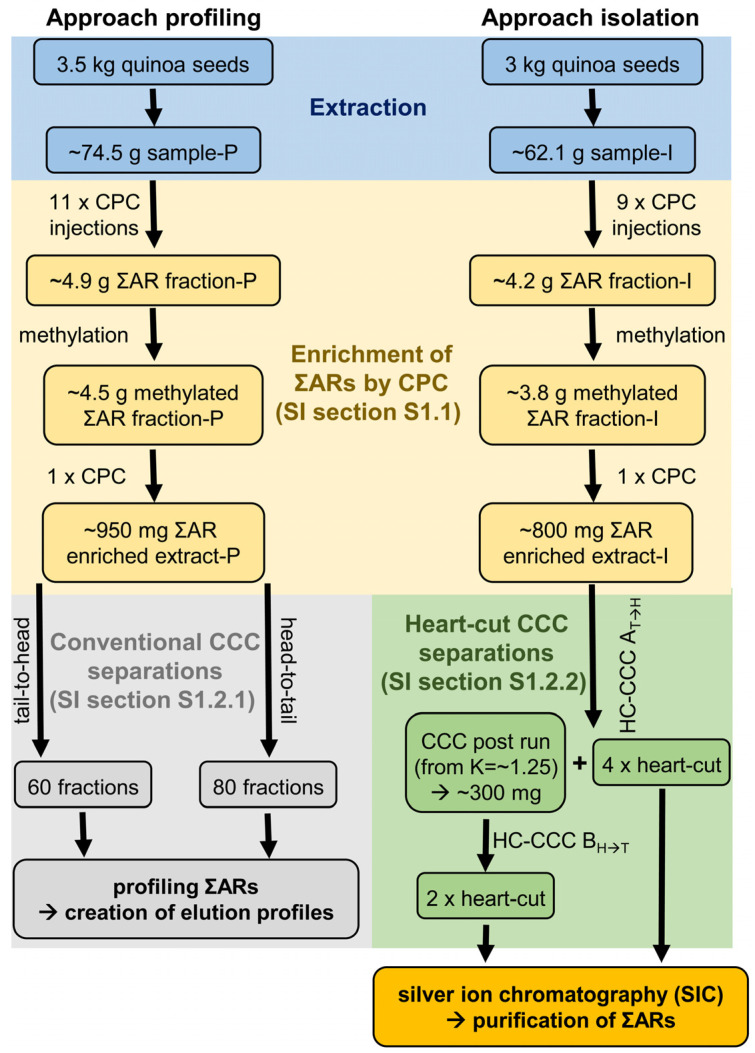
Flow chart of the sample preparation for both CCC approaches (conventional and heart-cut separations) for profiling and isolation of (methyl)alkylresorcinols from quinoa seeds.

Batches of 500 g whole quinoa seeds each were cold extracted with 400 mL of the azeotropic mixture of cyclohexane/ethyl acetate (CE, 46:54, *w*/*w*) with occasional shaking for 3 days at room temperature. In total, 3.5 kg (7 × 500 g) of quinoa seeds were extracted for two conventional CCC separations for AR profiling (sample-P, Section 3.4.1) and 3.0 kg (6 × 500 g) for HC-CCC separations for AR isolation (sample-I, Section 3.4.2, Figure 8).

The extractants were separated from the quinoa seeds using a folded filter and concentrated using rotary evaporation. Seven batches of sample-P and six batches of sample-I, respectively, were combined by transferring the resulting raw extracts with CE into pre-weighed 250 mL flasks. After removing the solvent by rotary evaporation, the masses of the combined extracts of sample-P and sample-I were determined gravimetrically.

### 3.3. Enrichment of Alkylresorcinols (ΣARs) via Centrifugal Partition Chromatography (CPC)

Aliquots of sample-P and sample-I were first enriched by CPC using a 250 PRO instrument (Gilson, Middleton, WI, USA) with the setup described in Hammerschick and Vetter [33]. The solvent system *n*-hexane/MeCN (1:1, *v*/*v*) was operated in ascending mode with a mobile phase flow rate of 5 mL/min at a rotor speed of 1600 rpm (S_f_ = 84–86%). With the exception of the first CPC run (which was fractionated in the same way), two injections were always made for one fractionation. Namely, from the 2nd injection on, a 2nd injection was made with an offset of 7 min or 35 mL (1st injection at 0 mL and 2nd injection at 35 mL). From these (one or) two injections, the ∑AR fraction (partition coefficients between lower and upper phase of AR: K_L/U_ ~1.8–17 (AR25:0-AR15:0)) was collected 250 mL after the beginning of the run via elution extrusion (CPC post run) with 300 mL methanol at a flow rate of 100 mL/min and a rotational speed of 500 rpm. In addition, the elution range 50–250 mL (10–50 min)—which corresponded with the elution range 15–215 mL of the 2nd injection—was collected just to determine the weight.

In total, eleven aliquots of sample-P (~74.5 g, average sample amount per injection ~6.8 g) were injected in six CPC runs and all post runs were concentrated and pooled to give the ∑AR fraction-P, which was used for the “approach profiling” in conventional CCC injections (Figure 8). In the same way, nine aliquots of sample-I (~62.1 g, average sample amount per injection ~6.9 g) were injected and post runs were combined to give the ∑AR fraction-I, which was used for the “approach isolation” in subsequent HC-CCC separations (Figure 8). Detailed information on CPC separations can be found in the supporting information (SI Section S1.1).

The ∑AR fraction-P (~4.9 g) and the ∑AR fraction-I (~4.2 g) were preparatively methylated with 1% sulphuric acid in methanol in order to transfer free fatty acids (FFAs) into fatty acid methyl esters (FAMEs) (Figure 8) [32]. The obtained methylated extracts (methylated ∑AR fraction-P: ~4.5 g (~92%); methylated ∑AR fraction-I: ~3.8 g (~90%)) were separated again by CPC in one injection, respectively, with the same setup described above (Figure 8). In this step, FAMEs eluted during the first 250 mL could be separated from ΣARs, which were again collected by elution extrusion with methanol (300 mL, ∑AR enriched extract-P/I).

### 3.4. Isolation of Alkylresorcinols (ARs) by Countercurrent Chromatography (CCC)

CCC separations were performed with the biphasic solvent system *n*-hexane/ethyl acetate/methanol/water (9:1:9:1, *v*/*v*/*v*/*v*, HEMWat-7) [48] on a QuikPrep MK8 instrument (AECS, London, UK) [44]. Separations were performed with a mobile phase flow rate of 2 mL/min and a maximum rotor speed of 870 rpm. Detailed information on CCC separations can be found in SI (SI Section S1.2).

#### 3.4.1. Conventional CCC Separations (with Pool Approach Profiling)

Two conventional CCC separations using coil 2 + 3 (tube volume 236 mL) were performed with the ∑AR enriched extract-P. In tail-to-head mode, ~300 mg of the dissolved sample was separated with the equilibrated CCC system (S_f_ = 86%, “conventional CCC 1_T→H_”, Figure 8). After a pre-run of 80 mL, 60 fractions of 7 mL each were collected. For the “conventional CCC 2_H→T_” separation in head-to-tail mode, 650 mg dissolved sample of the ∑AR-enriched extract-P was injected into the equilibrated CCC system (S_f_ = 86%). In this case, 80 fractions of 7 mL each were collected after a pre-run of 80 mL (Figure 8).

#### 3.4.2. Heart-Cut CCC Separations (with Pool Approach Isolation)

For the HC-CCC separation, two different separation systems (dimensions) are required, which in this case consisted of interconnected coils 2 + 3 (1st dimension, 236 mL) and coils 1 + 4 (2nd dimension, 235 mL). In the first HC-CCC separation (“HC-CCC A_T→H_”), which was performed in tail-to-head mode, ~800 mg of the ∑AR enriched extract-I was separated. Some selected ΣARs were isolated with four HCs (Figure 8). Finally, the CCC post run was collected at K = ~1.25 by elution extrusion with methanol.

More polar selected ΣARs were isolated in second HC-CCC separation (“HC-CCC B_H→T_”) in head-to-tail mode with two HCs, using the evaporated post run of the HC-CCC A_T→H_ separation as the sample (Figure 8). Detailed information on HC-CCC separations about the individual HCs can be found in Table 2 and in SI Section S1.2.2.

#### 3.4.3. Processing of CCC Fractions

CCC fractions were processed according to Hammerschick and Vetter [33]. In brief, CCC fractions were analyzed with GC/MS (Section 3.5) after removing the solvent via evaporation, the determination of the weights of the residues and silylating an aliquot. Using the GC/MS results of the saturated ΣARs only (without impurities), elution profiles were created.

### 3.5. Purification of (Methyl) Alkylresorcinols ((m)ARs) by Silver Ion Chromatography

Saturated mARs and ARs were purified by SIC separation according to the protocol of Hammerschick et al. [21] with slight modifications. In brief, aliquots of ~10–80 mg dissolved in 2 mL *n*-hexane/ethyl acetate (96:4, *v*/*v*) of the pooled HC-CCC fractions were placed onto conditioned columns. Typically, samples were subdivided into five SIC-fractions: (i) 50 mL *n*-hexane/ethyl acetate (96:4, *v*/*v* SIC-1), (ii, iii) 2 × 50 mL *n*-hexane/ethyl acetate (92:8, *v*/*v*, SIC-2.1 and SIC-2.2), (iv) 50 mL *n*-hexane/ethyl acetate (80:20, *v*/*v*, SIC-3), and finally (v) 50 mL ethyl acetate (SIC-4). In some cases, fractions SIC-2.1 and SIC-3 were further divided into 5 sub-fractions of 10 mL each. After the removal of the solvent and determination of the fraction weights, aliquots of each SIC fraction were analyzed by GC/MS post-silylation.

### 3.6. Gas Chromatography with Mass Spectrometry (GC/MS)

Aliquots of the CPC, CCC, and SIC fractions were silylated (50 µL BSTFA, 25 µL pyridine, 60 °C, 30 min) and analyzed on a 30 m Optima 5HT column in a 6890/5973 GC/MS system (Hewlett-Packard/Agilent, Waldbronn, Germany) using the parameters as described by Hammerschick et al. [21]. Compounds were identified via GC retention times and mass spectra according to Hammerschick et al. [17,21]. In particular, silylated ARs were detected by the extraction of the diagnostic fragment ions of the base peaks at *m*/*z* 282 (mARs) or *m*/*z* 268 (ARs), which are formed by McLafferty rearrangement of the di-fold trimethylsilylated (methylated) resorcinol ring [17,20,21].

### 3.7. NMR Spectroscopy

Proton (^1^H) spectra of each isolated compound were recorded on a 600 MHz Bruker Avance III spectrometer at 600 MHz. The samples were dissolved in deuterated methanol (*d*_4_-MeOH) and measured with a 5 mm BBO probe head. The spectra were calibrated with the residual solvent peak MeOH at δ 3.31 ppm as a reference.

## 4. Conclusions

The liquid–liquid chromatography technique of CPC was ideally suited as an instrument for sample preparation due to its high sample capacity. The quinoa extract (~75 g, ~62 g) was highly enriched in ΣARs (~0.95 g, ~0.8 g) by separating the more non-polar compounds, such as triacylglycerols, sterols, and FAMEs in 6–7 CPC runs, using the solvent system *n*-hexane/acetonitrile (1:1, *v*/*v*). In particular, the injection of two samples (2 × ~6–9 g) in short succession into the same equilibrated CPC system was very effective in terms of time (~70 min for complete separation) and resources (~300 mL each of lower phase, upper phase, and methanol) saved per injection. On the one hand, following conventional CCC was very effective in fractionating and enriching the individual ΣARs, resulting in the subsequent detection of 112 ΣARs, of which 63 had not been described yet. On the other hand, the potential of HC-CCC was used to further separate individual ΣARs, which was necessary for further purification due to the high number and diversity of ΣARs. A special feature was the time- and solvent-saving simultaneous separation in the second dimension of the HC-CCC setup of two elution ranges (HCs) transferred from the first to the second dimension. The application of column chromatography with silica gel coated with silver ions enabled the separation of saturated mARs from ARs, which was used for the isolation and purification of ten different ΣARs after the pooling of selected HC-CCC fractions. ΣARs standard substances in *iso*- and *anteiso*-configuration, which were not commercially available, were isolated up to double-digit mg levels and to >98% purity. Although the final isolation of ΣARs necessitated the subsequent application of SIC, the initial HC-CCC was required for the enrichment and pre-purification of the individual ΣARs from an ΣAR mixture of 112 different ΣARs. The final screening on ARs was carried out by GC/MS after the silylation of the fractions. As an alternative, LC-MS, similarly to Knödler et al. [49] or Kowalska and Jędrejek [50], may be used in the future as this method allows the omission of the silylation step.

## Figures and Tables

**Table 1:** Known and hitherto unknown alkylresorcinols and methylalkylresorcinols in quinoa seeds identified by GC/MS post-silylation after conventional CCC fractionation.

Alkylresorcinol (AR) ^a^	*m*/*z* [M]^+^	GC t_R_ [min]	Contribution to ΣARs	K Value ^b^	Methylalkylresorcinol (mAR) ^c^	*m*/*z* [M]^+^	GC t_R_ [min]	Contribution to ΣARs	K Value ^b^
**Saturated**					**Saturated**				
AR16:0	478	22.94	tr	0.31					
AR17:0 *	492	24.57	tr	0.40	mAR17:0 *	506	25.50	tr	0.54
AR18:0 *	506	26.31	tr	0.51	mAR18:0	520	27.28	tr	0.65
AR19:0 *	520	28.20	3.4%	0.65	mAR19:0 *	534	29.28	1.0%	0.82
AR20:0 *	534	30.14	0.3%	0.82	mAR20:0	548	31.27	0.1%	1.07
AR21:0 *	548	32.11	11.0%	1.06	mAR21:0 *	562	33.27	2.2%	1.37
AR22:0 *	562	34.09	0.5%	1.33	mAR22:0	576	35.26	0.1%	1.75
AR23:0 *	576	36.10	2.5%	1.71	mAR23:0 *	590	37.28	0.4%	2.16
AR24:0 *	590	38.07	tr	2.11	mAR24:0	604	39.27	tr	2.78
AR25:0 *	604	40.04	0.3%	2.61	mAR25:0	618	41.26	tr	^d^
AR26:0 *	618	42.00	tr	^d^					
AR15:0-*i*	464	21.47	tr	0.28	mAR15:0-*i*	478	22.16	tr	0.33
AR17:0-*i* *	492	23.93	tr	0.39	mAR17:0-*i*	506	24.81	tr	0.51
AR19:0-*i* *	520	27.47	2.3%	0.61	mAR19:0-*i* *	534	28.55	1.4%	0.78
AR20:0-*i*	534	29.36	tr	0.75	mAR20:0-*i*	548	30.51	tr	0.96
AR21:0-*i* *	548	31.36	14.3%	0.99	mAR21:0-*i* *	562	32.52	5.2%	1.30
AR22:0-*i* *	562	33.32	tr	1.23	mAR22:0-*i*	576	34.51	tr	1.61
AR23:0-*i* *	576	35.34	9.5%	1.61	mAR23:0-*i* *	590	36.54	2.4%	2.06
AR24:0-*i* *	590	37.29	tr	2.02	mAR24:0-*i*	604	38.54	tr	2.61
AR25:0-*i* *	604	39.32	1.5%	2.51	mAR25:0-*i* *	618	40.52	tr	^d^
AR26:0-*i*	618	41.27	tr	^d^					
AR27:0-*i*	632	43.38	tr	^d^					
AR16:0-*a*	478	22.55	tr	0.30					
AR18:0-*a* *	506	25.83	0.2%	0.47	mAR18:0-*a* *	520	26.85	0.1%	0.61
AR19:0-*a* *	520	27.59	tr	0.61	mAR19:0-*a* *	534	28.65	tr	0.79
AR20:0-*a* *	534	29.62	9.0%	0.75	mAR20:0-*a* *	548	30.76	3.9%	0.96
AR21:0-*a* *	548	31.54	tr	0.99	mAR21:0-*a* *	562	32.74	tr	1.30
AR22:0-*a* *	562	33.59	12.4%	1.23	mAR22:0-*a* *	576	34.78	4.9%	1.61
AR23:0-*a* *	576	35.51	tr	1.58	mAR23:0-*a* *	590	36.78	tr	2.06
AR24:0-*a* *	590	37.58	4.2%	1.96	mAR24:0-*a* *	604	38.79	0.9%	2.51
AR25:0-*a*	604	39.57	tr	2.44					
AR26:0-*a* *	618	41.52	0.2%	^d^	mAR26:0-*a* *	632	42.75	tr	^d^
**Monoenoic**					**Monoenoic**				
					mAR17:1	504	25.05	tr	0.33
AR19:1 *	518	27.71, 27.89	0.1%	0.44	mAR19:1	532	28.76, 28.96	tr	0.54
AR20:1	532	29.72	tr	0.54					
AR21:1 *	546	31.62, 31.81	2.2%	0.68	mAR21:1 *	560	32.83, 33.00	0.5%	0.85
AR22:1 *	560	33.65	tr	0.82					
AR23:1 *	574	35.70, 35.84	0.5%	1.06	mAR23:1	588	36.85, 37.05	0.1%	1.40
AR25:1	602	39.68	tr	1.71	mAR25:1	616	40.91	tr	2.16
AR27:1	630	43.87	tr	2.68					
AR21:1-*i*	546	30.73, 30.88	tr	0.61	mAR21:1-*i*	560	32.06	tr	0.78
AR20:1-*a*	532	29.07	tr	0.54					
AR22:1-*a*	560	33.06	0.2%	0.75	mAR22:1-*a* *	574	34.28	0.1%	1.02
AR24:1-*a*	588	37.12	tr	1.23	mAR24:1-*a*	602	38.30	tr	1.58
AR26:1-*a*	616	41.10	tr	1.92					
**Dienoic**					**Dienoic**				
AR17:2	488	27.55	tr	0.30					
					mAR19:2	530	28.74	tr	0.40
AR21:2 *	544	31.60	0.5%	0.33	mAR21:2	558	32.79	0.1%	0.61
AR22:2	558	33.47	tr	0.47					
AR23:2 *	572	35.66	0.1%	0.75	mAR23:2	586	36.89	tr	0.99
AR25:2	600	39.63	tr	1.16					
**Trienoic**					**Trienoic**				
AR21:3	542	31.82	tr	0.33					
**Keto group**					**Keto group**				
AR19:0 oxo	534	30.51	tr	0.27	mAR19:0 oxo	548	31.46	tr	0.32
AR21:0 oxo	562	34.54	tr	0.33					
AR23:0 oxo	590	38.53	tr	0.51					
					mAR19:0-*i* oxo	548	30.80	tr	0.31
AR21:0-*i* oxo	562	33.64	tr	0.33	mAR21:0-*i* oxo	576	34.78	tr	0.51
AR23:0-*i* oxo	590	37.75	tr	0.51					
AR25:0-*i* oxo	618	41.76	tr	0.82					
AR20:0-*a* oxo	548	31.94	tr	0.30	mAR20:0-*a* oxo	562	33.04	tr	0.37
AR22:0-*a* oxo	576	36.00	tr	0.37	mAR22:0-*a* oxo	590	37.12	tr	0.58
AR24:0-*a* oxo	604	39.98	tr	0.58					
AR23:1 oxo	588	38.1	tr	0.33					

^a^ characteristic base ion *m*/*z* 268; ^b^ K values of the ∑ARs were determined using the maxima of the elution volumes of the conventional CCC 2_H→T_ run performed in head-to-tail mode; ^c^ characteristic base ion *m*/*z* 282; ^d^ elution of the respective ∑ARs in the CCC post run; “tr” indicates trace amounts, i.e., contributions <0.1% to the total ΣAR content; and * is known ∑ARs in the literature [14,15,18].

**Table 2:** Intended heart-cut ranges based on the elution profiles from the conventional CCC separations together with the actually used transfer ranges of the separation in the first dimension for the respective heart-cuts and the fractionation ranges of the heart-cut separation in the second dimension.

	1st Dimension		2nd Dimension
**Heart-Cut**	**Performed ^a^ (Intended ^b^) Transfer Range [mL]**	**Targeted AR** **(Purities)**	**Purities of the Target AR in the Pooled Heart-Cut Fractions for SIC ^c^**
A_T→H_, HC 1	132–151 (123–151)	mAR23:0-*i* (22%) AR24:0-*a* (38%)	mAR23:0-*i* ^d^ AR24:0-*a* (58%, 144–164 mL)
A_T→H_, HC 2	151–173 (151–182)	mAR22:0-*a* (26%) AR23:0-*i* (37%)	mAR22:0-*a* (45%, 153–174 mL) AR23:0-*i* ^d^
A_T→H,_ HC 3	188–212 (197–221)	mAR21:0-*i* (18%) AR22:0-*a* (58%)	mAR21:0-*i* (25%, 307–328 mL) AR22:0-*a* (60%, 230–251 mL)
A_T→H,_ HC 4	212–289 (221–280)	mAR20:0-*a* (15%) AR21:0-*i* (48%)	mAR20:0-*a* (22%, 244–307 mL) AR21:0-*i* ^d^
B_H→T,_ HC 1	134–156 (136–157)	mAR18:0-*a* (2%) AR19:0-*i* (45%)	mAR18:0-*a* (3%, 134.5–169.5) AR19:0-*i* ^d^
B_H→T,_ HC 2	172–204.5 (172–205)	mAR19:0-*i* (11%) AR20:0-*a* (65%)	mAR19:0-*i* (11%, 239.5–269.5 mL) AR20:0-*a* (88%, 239.5–269.5 mL)

^a^ actual transfer ranges of the performed heart-cuts, only consideration of elution volume in 1st dimension; ^b^ intended transfer ranges for the heart-cuts, elution volumes were selected based on the elution profiles of the conventional CCC separation; ^c^ elution volume for fractionation flowing through the 2nd dimension. Starting point of the transfer of the heart-cut range was set to 0 mL. ^d^ no economically effective SIC fractionation.

**Table 3:** Masses and purities of the (methyl)alkylresorcinols ((m)AR) purified by silver ion chromatography (SIC) from the respective heart-cut CCC elution ranges.

Isolated (m)AR	Mass [mg]	Purity [%]	CCC Elution Volumes Used for SIC
mAR18:0-*a*	0.6	>97%	B_H→T_, HC 1 + 2: 134.5–169.5 mL
mAR19:0-*i*	0.9	>98%	B_H→T_, HC 1 + 2: 239.5–269.5 mL
mAR20:0-*a*	15.5	>97%	A_T→H_, HC 2 + 3: 349–426 mL, HC 4: 244–307 mL
mAR21:0-*i*	1.7	>93%	A_T→H_, HC 2 + 3: 307–328 mL, HC 4: 202–223 mL
mAR22:0-*a*	15.3	>96%	A_T→H_, HC 1: 184–209 mL, HC 2 + 3: 153–174 mL
mAR24:0-*a*	2.4	>95%	A_T→H_, 104–114 mL
AR20:0-*a*	7.5	>96%	B_H→T_, HC 1 + 2, 239.5–269.5 mL
AR21:0-*i*	3.0	>90%	A_T→H_, HC 1: 321–342 mL; B_H→T_, HC 1 + 2: 309.5–339.5 mL
AR22:0-*a*	13.0	>95%	A_T→H_, HC 2 + 3: 230–251 mL, 279–293 mL, HC 4: 188–216 mL
AR24:0-*a*	3.8	>95%	A_T→H_, HC 1: 144–164 mL

## Data Availability

The data presented in this study are available on request from the corresponding author.

## Supplementary Materials

The following supporting information can be downloaded at: https://www.mdpi.com/article/10.3390/molecules28135220/s1, Figure S1: Elution profiles of only the saturated (**A**) total alkylresorcinols, (**B**) methyl alkylresorcinols (mAR), and (**C**) the alkylresorcinols (AR) of the conventional CCC 1_T→H_ separation in tail-to-head mode with the solvent system *n*-hexane/ethyl acetate/methanol/water (9:1:9:1, *v*/*v*/*v*/*v*); Figure S2: Elution profiles of only the saturated (**A**) total alkylresorcinols, (**B**) methyl alkylresorcinols (mAR), and (**C**) the alkylresorcinols (AR) of the conventional CCC 2_H→T_ separation in head-to-tail mode with the solvent system *n*-hexane/ethyl acetate/methanol/water (9:1:9:1, *v*/*v*/*v*/*v*); Figure S3: (**A**) Excerpt of the elution profiles of only the saturated total alkylresorcinols, methyl alkylresorcinols (mAR), and alkylresorcinols (AR) of the conventional CCC 1_T→H_ separation in tail-to-head mode with marked elution ranges (heart-cut 1—heart-cut 4) intended for transfer from the 1st to the 2nd dimension during HC-CCC A_T→H_. Elution profiles of only the saturated total alkylresorcinols, methyl alkylresorcinols (mAR), and alkylresorcinols (AR) separated (together) in the 2nd dimension after transfer of (**B**) heart-cut 1, (**C**) heart-cut 2 and heart-cut 3, and (**D**) heart-cut 4 from the 1st dimension during HC-CCC A_T→H_ separation in head-to-tail mode. The elution volume passing through the 1st dimension up to the beginning of the heart-cut was added as a value in front of the x-axis; Figure S4: (**A**) Excerpt of the elution profiles of only the saturated total alkylresorcinols, methyl alkylresorcinols (mAR), and alkylresorcinols (AR) of the conventional CCC 2_H→T_ separation in head-to-tail mode with marked elution ranges (heart-cut 1 and heart-cut 2) intended for transfer from the 1st to the 2nd dimension during HC-CCC B_H→T_. (**B**) Elution profiles of only the saturated total alkylresorcinols, methyl alkylresorcinols (mAR), and alkylresorcinols (AR) separated together in the 2nd dimension after transfer of heart-cut 1 and heart-cut 2 elution ranges from the 1st dimension during HC-CCC B_H→T_ separation in head-to-tail mode. The elution volume passing through the 1st dimension up to the beginning of the heart-cut was added as a value in front of the x-axis. The entire elution range of the target analyte AR20:0-*a* and the range used for the subsequent SIC are marked in color; Figure S5: GC/MS Spectra of the TMS derivatives of the isolated compounds (**A**) mAR18:0-*a*, (**B**) mAR20:0-*a*, (**C**) mAR22:0-*a*, (**D**) mAR24:0-*a*, (**E**) mAR19:0-*i*, (**F**) mAR21:0-*i*, (**G**) AR20:0-*a*, (**H**) AR22:0-*a*, (**I**) AR24:0-*a*, and (**J**) AR21:0-*i*; Figure S6: ^1^H NMR spectra of the isolated compounds (**A**) mAR18:0-*a*, (**B**) mAR20:0-*a*, (**C**) mAR22:0-*a*, (**D**) mAR24:0-*a*, (**E**) mAR19:0-*i*, (**F**) mAR21:0-*i*, (**G**) AR20:0-*a*, (**H**) AR22:0-*a*, (**I**) AR24:0-*a*, and (**J**) AR21:0-*i*. The spectra were calibrated with the residual solvent peak MeOH (highlighted in green) at δ 3.31 ppm as a reference. In addition, the signal of the water contained in the deuterated solvent was marked in blue.; Table S1: ^1^H chemical shift assignments (*δ*_H_ [ppm]) and multiplicities for isolated mARs and ARs (deuterated methanol was used as solvent) compared with the literature data of AR19:0 of Hammerschick et al. [32].

## Sample Availability

Aliquots of the compounds may be requested from the authors.
