# Supplementary material for: Profiling and Isolation of Ten Rare Branched-Chain Alkylresorcinols in Quinoa
Source: Molecules. 2023 Jul 5;28(13):5220. doi: 10.3390/molecules28135220 (PMC10343731; doi:10.3390/molecules28135220)
Supplement: Supplementary file 1 [file molecules-28-05220-s001.zip › molecules-2421522-supplementary.pdf]

Supplementary information for manuscript:

Article related abbreviations

AR, alkylresorcinol; CCC, countercurrent chromatography; CPC, centrifugal partition chromatography; CE, azeotropic mixture of cyclohexane/ethyl acetate; d_4-MeOH, deuterated methanol; FFA, free fatty acid; FAME, fatty acid methyl ester; HC, heart-cut; ISTD, internal standard; mAR, 2-methylalkylresorcinol; ME, methyl ester; S_f, retention of the stationary phase; TMS, trimethylsilyl

1 Countercurrent separation

1.1 Enrichment of alkylresorcinols (ARs) by centrifugal partition chromatography (CPC)

Aliquots of sample-P and sample-I were first enriched by CPC using a 250 PRO instrument (Gilson, Middleton, WI, USA) with the setup described in Hammerschick and Vetter [1]. The solvent system *n*-hexane/MeCN (1:1, v/v) was operated in ascending mode. After equilibration of the solvent system (vigorous shaking of 500 mL *n*-hexane and 500 mL MeCN, phase separation after 30 min equilibration time, 10 min ultrasonication) and phase separation, the stationary (lower) phase was pumped with a flow rate of 100 mL/min into the CPC rotor maintained at 500 rpm. After 300 mL, the mobile (upper) phase was pumped into the system at 5 mL/min at a rotation speed of 1600 rpm. With the breakthrough of the mobile phase (displacement of 36-40 mL stationary phase, S_f = 84-86%), ~6-9 g quinoa seed extract, dissolved in both 10 mL lower and upper phase, was repeatedly injected via a 30 mL sample loop (first injection). With the exception of the first CPC run (which was fractionated in the same

way), always two injections were made for one fractionation. Namely, from the second injection on, a 2nd injection was made with an offset of 35 mL (first injection at 0 mL and second injection at 35 mL). In total, eleven samples (~74.5 g, average sample amount per injection ~6.8 g) of the extract "approach profiling" were injected and the post runs were pooled to give the ∑AR fraction-P which was used for the "approach profiling" in conventional CCC injections). In the same way, nine aliquots of sample-I (~62.1 g, average sample amount per injection ~6.9 g) were injected and post runs were combined to give the ∑AR fraction-I that was used for the "approach isolation" in subsequent heart-cut CCC separations.

The CPC chromatogram was monitored at 210 nm (flash 10 diode array detector, DAD, Ecom, Praha, Czech Republic) and the elution range 50-250 mL (10-50 min) after the first injection (corresponded to "15-215 mL" of the second injection) was entirely collected (CPC fraction 1) with a Gilson FC 204 fraction collector (Middleton, WI, USA). After 50 min (250 mL) of the first injection (corresponded to "215 mL" of the second injection), a further fraction ("∑AR fraction-P/I") of 300 mL was collected after the start of elution extrusion (CPC post run) with methanol at a flow rate of 100 mL/min and a rotational speed of 500 rpm. The solvent of the fractions, collected in 500 mL glass flasks was removed with a rotary evaporator and each fraction's weights were determined.

The ∑AR fraction-P (~4.9 g) and the ∑AR fraction-I (~4.2 g) were preparatively methylated with 1% sulphuric acid in methanol in order to transfer free fatty acids (FFAs) into fatty acid methyl esters (FAMEs) [2]. The obtained methylated extracts

(methylated ∑AR fraction-P: ~4.5 g (~92%); methylated ∑AR fraction-I: ~3.8 g (~90%)) were separated again by CPC in one injection, respectively, with the same setup described above. In this step, FAMEs eluted during the first 250 mL and could be separated from ΣARs, which were again collected by elution extrusion with methanol (300 mL, ∑AR enriched extract-P/I).

1.2 Isolation of alkylresorcinols (ARs) by countercurrent chromatography (CCC)

The standard setup of the QuikPrep MK8 instrument (AECS, London, UK) and in-house modifications were described in detail by Englert *et al.* [3] and Müller *et al.* [4]. The most important component of the CCC centrifuge is the two opposite, dynamically installed bobbins, each with two individual coils with a volume of ~120 mL (bobbin 1: coil 1 116 mL, coil 2 122 mL; bobbin 2: coil 3 114 mL, coil 4 119 mL).

CCC separations were performed with the biphasic solvent system *n*-hexane/ethyl acetate/methanol/water (9:1:9:1, *v*/*v*/*v*/*v*, HEMWat-7) [5]. The coils selected for the separation were filled with stationary phase at a flow rate of 10 mL/min. After reducing the flow rate to 2 mL/min and setting the maximum rotor speed of 870 rpm, the system in the respective combined coils (depending on the separation) was equilibrated with the mobile phase and the stationary phase retention (S_f value) was determined. The samples were always dissolved in 4.5 mL upper and 4.5 mL lower phase.

1.2.1 Conventional CCC separations

Two conventional CCC separations using coil 2 + 3 (tube volume 236 mL) were performed with the ∑AR enriched extract-P. In tail-to-head mode, ~300 mg of the dissolved sample was separated with the equilibrated CCC system (S_f = 86%, "conventional CCC $1_{T\rightarrow H}$"). After a pre-run of 80 mL, 60 fractions of 7 mL each were collected. For the "conventional CCC $2_{H\rightarrow T}$" separation using the head-to-tail mode, 650 mg dissolved sample of the ∑AR enriched extract-P was injected into the equilibrated CCC system (S_f = 86%). In this case, 80 fractions of 7 mL each were collected after a pre-run of 80 mL.

1.2.2 Heart-cut CCC separations

For the heart-cut CCC (HC-CCC) separation, two different separation systems (dimensions) are required, which in this case consisted of interconnected coils 2 + 3 (1st dimension, 236 mL) and coils 1 + 4 (2nd dimension, 235 mL). By means of special T-pieces, 6-port selection valves, and the peripheral setup of the CCC, a transfer of a special elution range from the 1st to the 2nd dimension was possible, as well as the individual separation of the 1st and 2nd dimension, which was described in detail by Rüttler *et al.* [6].

For the first HC-CCC separation ("HC-CCC $A_{T\rightarrow H}$") which was performed in tail-to-head mode, ~800 mg of the ∑AR enriched extract-I was injected into the equilibrated 1st dimension (S_f = 91%). After a first fractionation (44.5-66 min, 9 fractions of 5 mL each) of the separation in the 1st dimension, 19 mL (66-75.5 min) of the effluent from the 1st dimension was transferred to the second one (S_f = 90%, heart-cut 1). After

the transfer, the mobile phase was only pumped through the 2nd dimension to separate and fractionate (105.5-183 min, 31 fractions of 5 mL each) the transferred compounds of heart-cut 1 (HC 1). A second transfer of 22 mL (183-194 min) was pumped from the 1st to the 2nd dimension (HC 2). In the time range between 194-224 min, only the 2nd dimension was used for separation, before three fractions of 5 mL each were collected from 224-231.5 min after the separation of the 1st dimension. Then, an effluent of 24 mL (231.5-244.5 min) was transferred from the 1st to the 2nd dimension (HC 3). Only the 2nd dimension was used for separation and fractionation (254-411.5 min, 45 fractions of 7 mL each) of the analytes transferred in HC 2 and HC 3. A final transfer of 77 mL (411.5-450 min) from the 1st to the 2nd dimension was conducted (HC 4). Separation and fractionation (502.5-660 min, 45 fractions of 7 mL each) of the analytes from HC 4 were performed only using the 2nd dimension. The analytes remaining in the 1st (from K=~1.25) and the 2nd dimension were eluted from the coils by elution extrusion with methanol at a flow rate of 10 mL/min (CCC post run).

For the second HC-CCC separation ("HC-CCC $B_{T\rightarrow H}$") using the head-to-tail mode, the post run of the HC-CCC $A_{T\rightarrow H}$ separation was evaporated to dryness (~300 mg), dissolved in 4.5 mL upper and 4.5 mL lower phase and injected into the 1st dimension (S_f = 85%). Between the time range of 40-67 min after injection, 10 fractions of 5 mL each were collected with the effluent from the 1st dimension. Then, for HC 1, 22 mL (67-78 min) was transferred from the effluent of the 1st to the 2nd dimension. Only the 2nd dimension was used for separation during 78-108 min, before only the 1st dimension was used to separate and fractionate (108-116 min, 4 fractions of 5 mL each)

analytes remaining in the 1st dimension. A second heart-cut (HC 2) transferring 32.5 mL (116-132.25 min) of the effluent from the 1st to the 2nd dimension was performed, immediately followed by the separation and fractionation (132.23-257.25 min, 50 fractions of 5 mL each) of the 2nd dimension only.

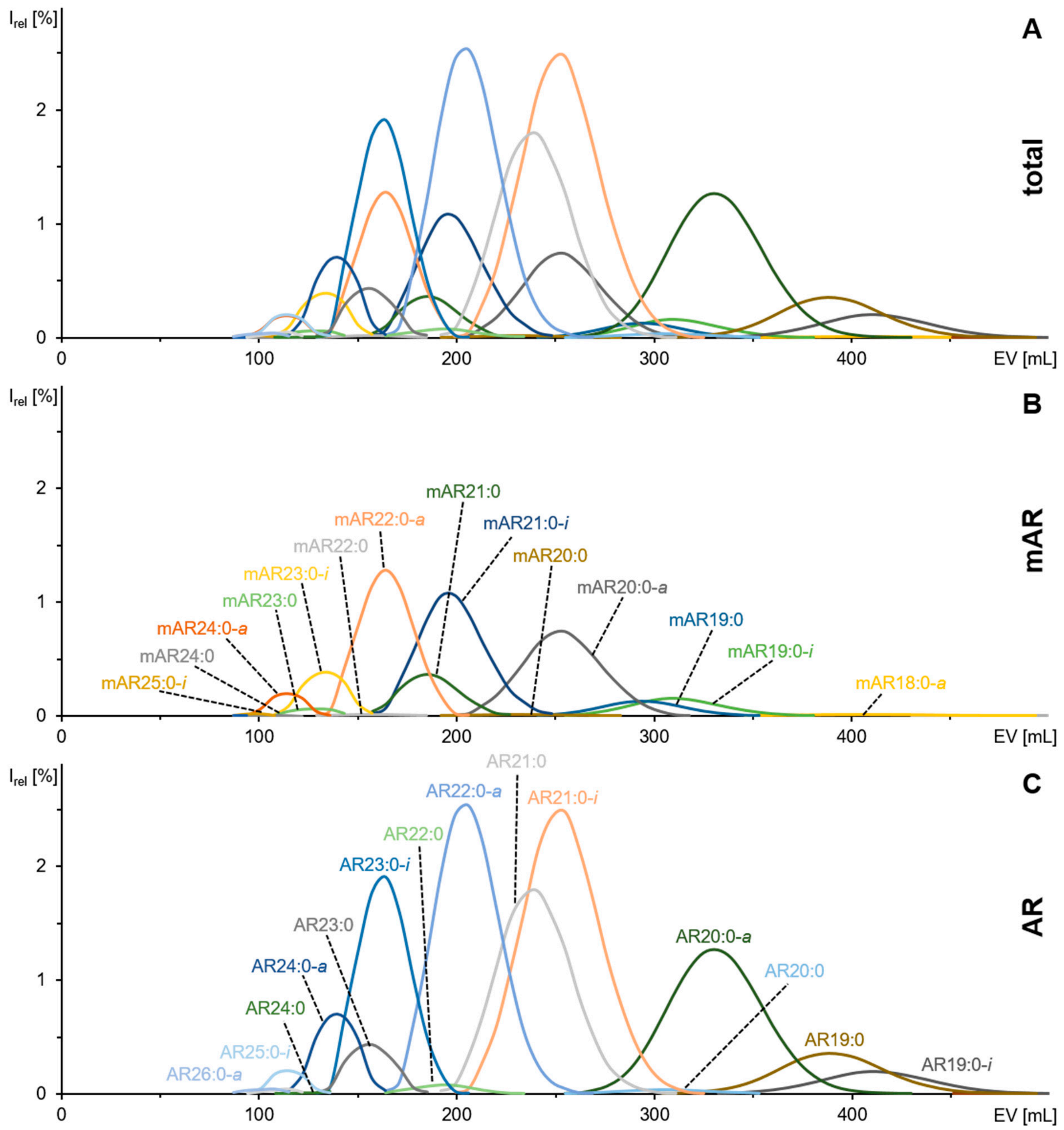

Figure S1 Elution profiles of only the saturated (**A**) total alkylresorcinols, (**B**) methyl alkylresorcinols (mAR), and (**C**) the alkylresorcinols (AR) of the conventional CCC $1_{T \rightarrow H}$ separation in tail-to-head mode with the solvent system *n*-hexane/ethyl acetate/methanol/water (9:1:9:1, v/v/v/v).

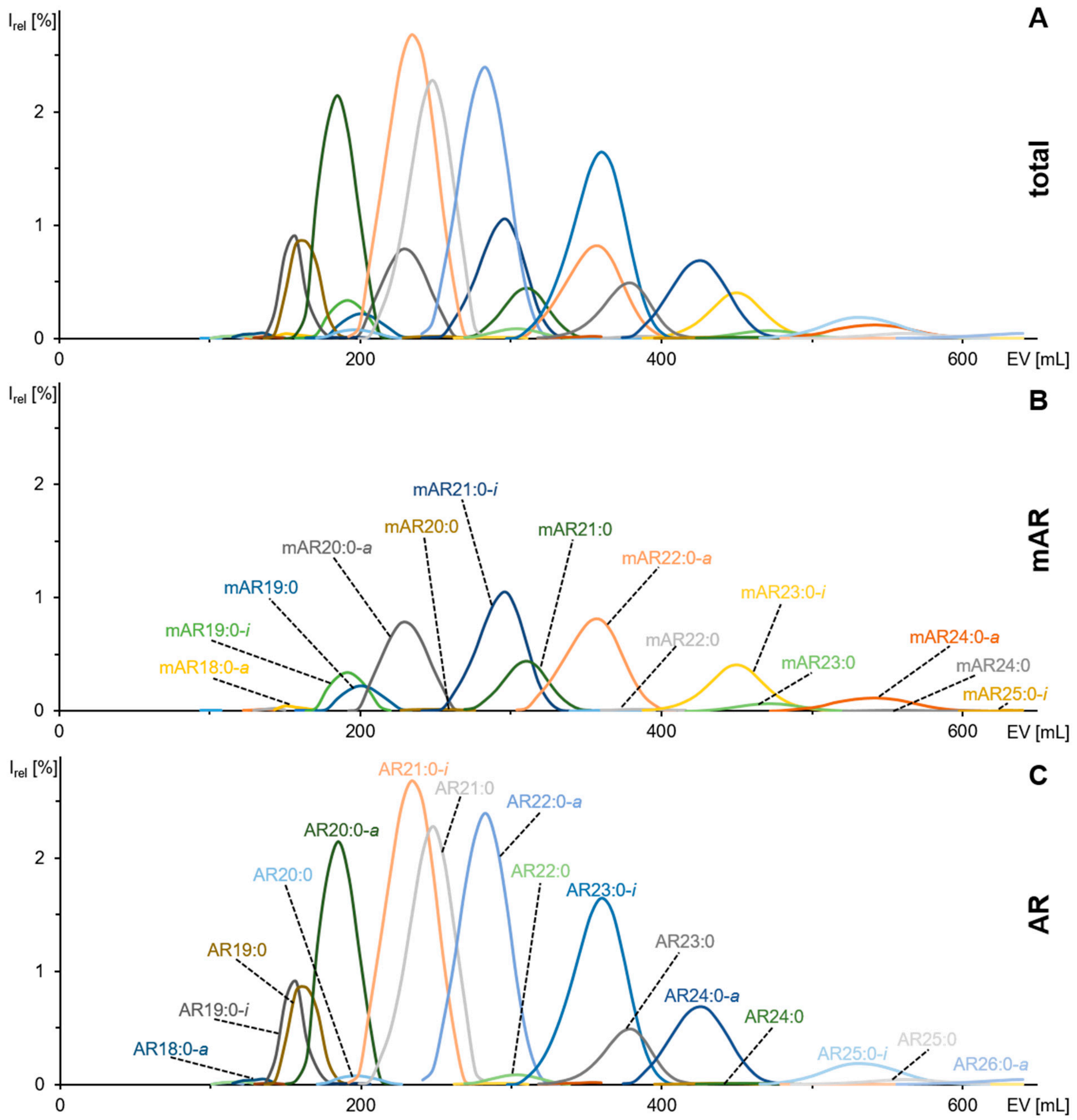

Figure S2 Elution profiles of only the saturated (**A**) total alkylresorcinols, (**B**) methyl alkylresorcinols (mAR), and (**C**) the alkylresorcinols (AR) of the conventional CCC $2_{H \rightarrow T}$ separation in head-to-tail mode with the solvent system *n*-hexane/ethyl acetate/methanol/water (9:1:9:1, v/v/v/v).

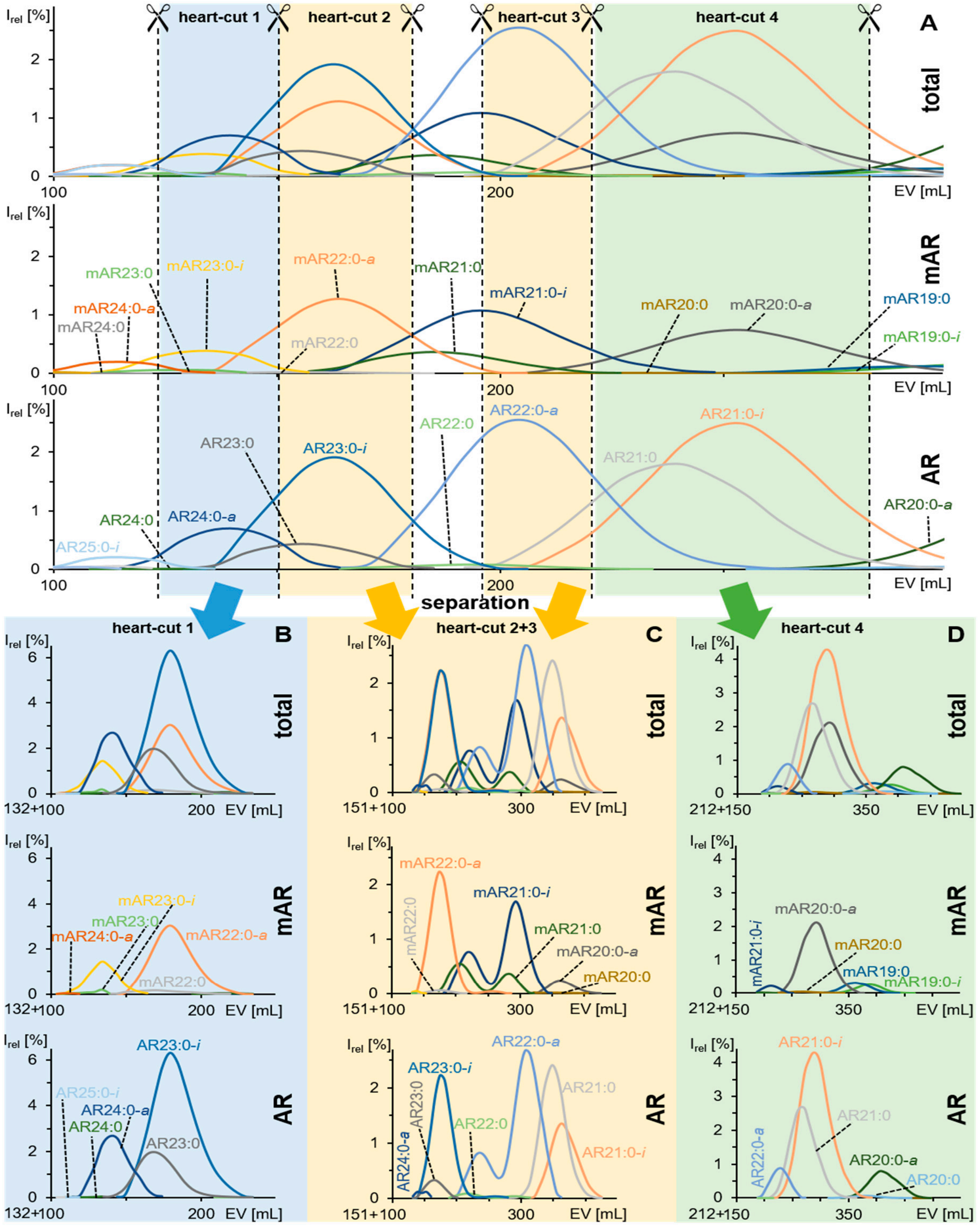

Figure S3 (A) Excerpt of the elution profiles of only the saturated total alkylresorcinols, methyl alkylresorcinols (mAR), and alkylresorcinols (AR) of the conventional CCC $1_{T\rightarrow H}$ separation in tail-to-head mode with marked elution ranges (heart-cut 1 – heart-cut 4) intended for transfer from the 1st to the 2nd dimension during HC-CCC $A_{T\rightarrow H}$. Elution profiles of only the saturated total alkylresorcinols, methyl alkylresorcinols (mAR), and alkylresorcinols (AR) separated (together) in the 2nd dimension after transfer of **(B)** heart-cut 1, **(C)** heart-cut 2 and heart-cut 3, and **(D)** heart-cut 4 from the

1st dimension during HC-CCC $A_{T \rightarrow H}$ separation in head-to-tail mode. The elution volume passing through the 1st dimension up to the beginning of the heart-cut, was added as a value in front of the x-axis.

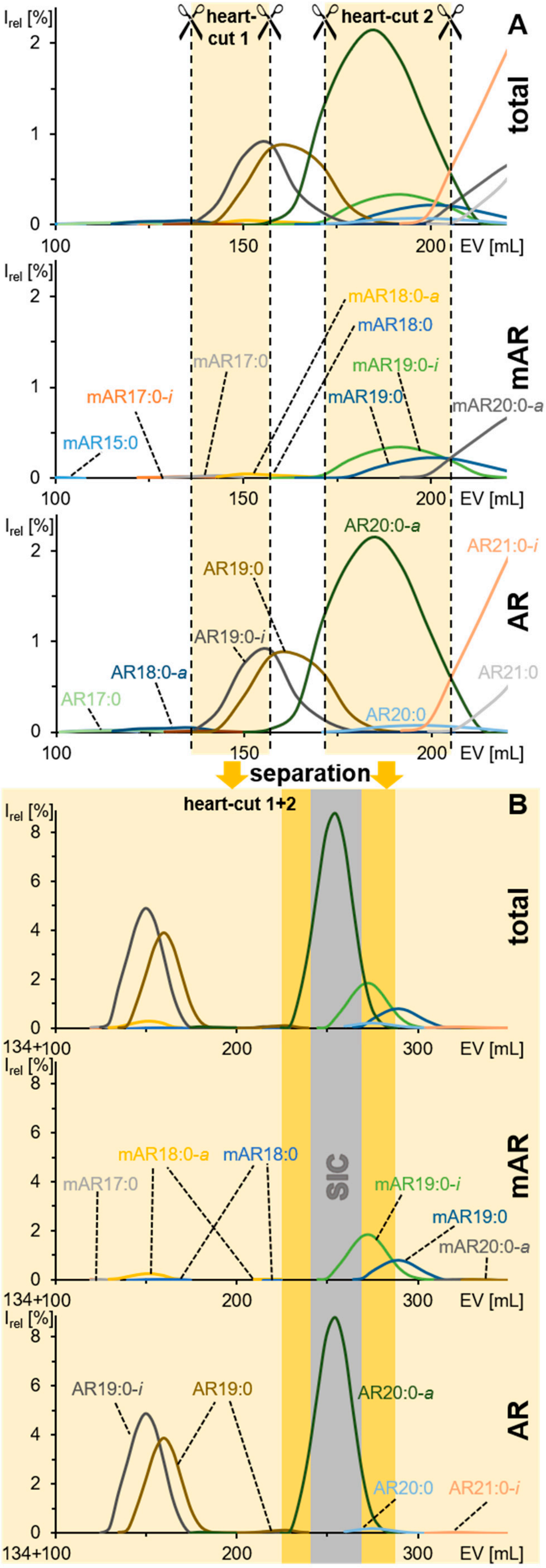

Figure S4 **(A)** Excerpt of the elution profiles of only the saturated total alkylresorcinols, methyl alkylresorcinols (mAR), and alkylresorcinols (AR) of the conventional CCC $2_{H\rightarrow T}$ separation in head-to-tail mode with marked elution ranges (heart-cut 1 and heart-cut 2) intended for transfer from the 1st to the 2nd dimension during HC-CCC $B_{H\rightarrow T}$. **(B)** Elution profiles of only the saturated total alkylresorcinols, methyl alkylresorcinols (mAR), and alkylresorcinols (AR) separated together in the 2nd dimension after transfer of heart-cut 1 and heart-cut 2 elution ranges from the 1st dimension during HC-CCC $B_{H\rightarrow T}$ separation in head-to-tail mode. The elution volume passing through the 1st dimension up to the beginning of the heart-cut, was added as a value in front of the x-axis. The entire elution range of the target analyte AR20:0-*a* and the range used for the subsequent SIC are marked in colour.

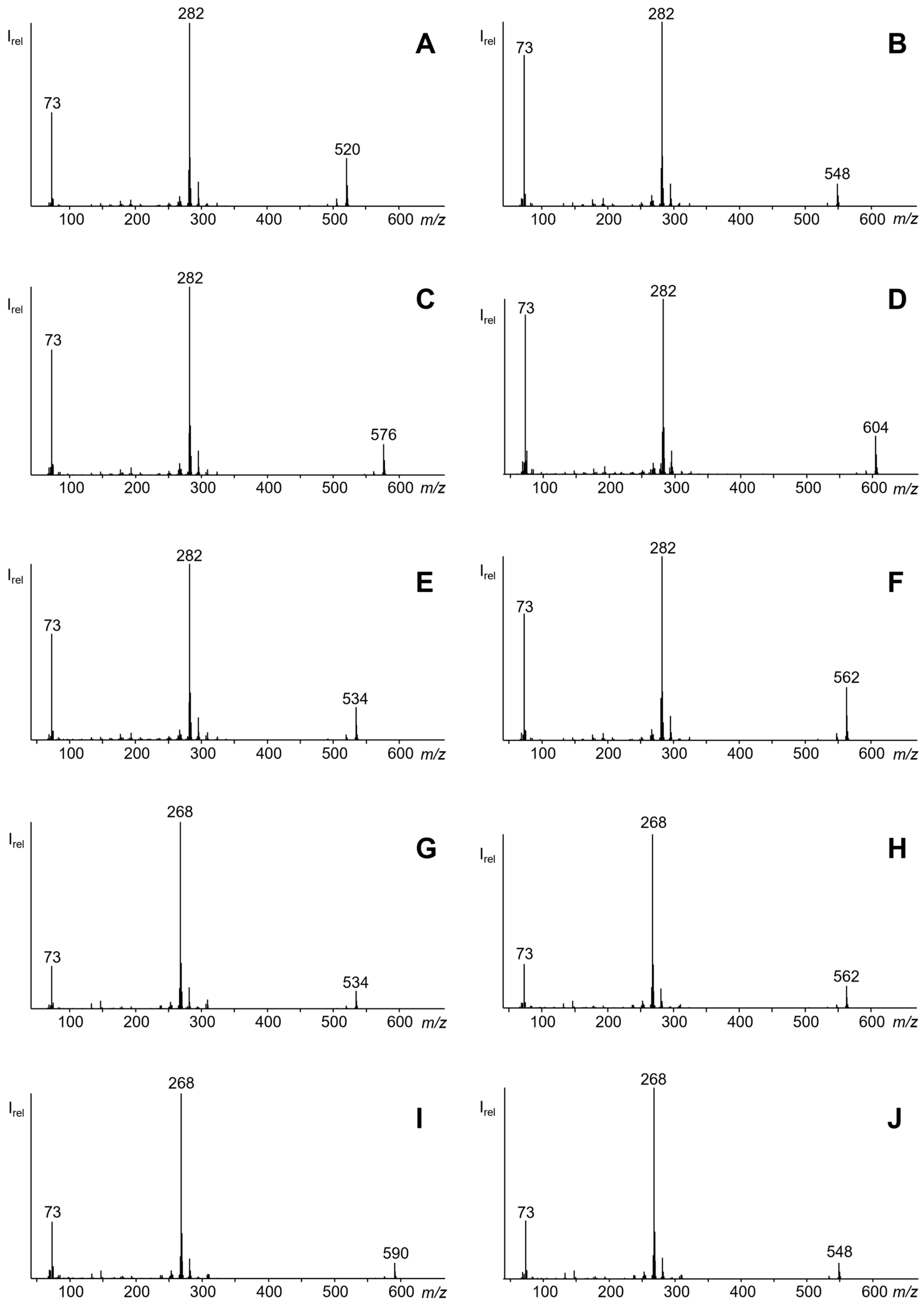

Figure S5 GC/MS Spectra of the TMS derivatives of the isolated compounds (**A**) mAR18:0-*a*, (**B**) mAR20:0-*a*, (**C**) mAR22:0-*a*, (**D**) mAR24:0-*a*, (**E**) mAR19:0-*i*, (**F**) mAR21:0-*i*, (**G**) AR20:0-*a*, (**H**) AR22:0-*a*, (**I**) AR24:0-*a*, and (**J**) AR21:0-*i*.

Table S1 ^{1}H NMR chemical shift assignments (δ_H [ppm]) and multiplicities for isolated ARs and mARs (deuterated methanol was used as solvent) compared with literature data of AR19:0 of Hammerschick *et al.* [2].

Position	mAR18:0-*a*	mAR20:0-*a*	mAR22:0-*a*	mAR24:0-*a*	mAR19:0-*i*	mAR21:0-*i*	AR20:0-*a*	AR22:0-*a*	AR24:0-*a*	AR21:0-*i*	AR19:0 [1]
2							6.07, t	6.07, t	6.07, t	6.07, t	6.08, t
2-CH_3	1.99, s	1.99, s	1.99, s	1.99, s	1.99, s	1.99, s					
4	6.14, s	6.14, s	6.14, s	6.14, s	6.14, s	6.14, s	6.12, d	6.12, d	6.12, d	6.12, d	6.12, d
6	6.14, s	6.14, s	6.14, s	6.14, s	6.14, s	6.14, s	6.12, d	6.12, d	6.12, d	6.12, d	6.12, d
1´	2.40, t	2.40, t	2.40, t	2.40, t	2.40, t	2.40, t	2.43, t	2.43, t	2.43, t	2.43, t	2.43, t
2´	1.55, qi	1.55, qi	1.55, qi	1.55, qi	1.55, qi	1.55, qi	1.56, qi	1.56, qi	1.56, qi	1.56, qi	1.56, qi
3´- 15´	1.28, m	1.28, m	1.29, m	1.29, m	1.29, m	1.29, m	1.29, m	1.29, m	1.29, m	1.29, m	1.29, m
15´-CH_3	0.86, d										
16´	1.28, m	1.28, m	1.29, m	1.29, m	1.29, m	1.29, m	1.29, m	1.29, m	1.29, m	1.29, m	1.29, m
17´	0.87, t	1.28, m	1.29, m	1.29, m	1.29, m	1.29, m	1.29, m	1.29, m	1.29, m	1.29, m	1.29, m
17´-CH_3		0.86, d			0.88, d		0.86, d				
18´		1.28, m	1.29, m	1.29, m	0.88, d	1.29, m	1.29, m	1.29, m	1.29, m	1.29, m	1.29, m
19´		0.87, t	1.29, m	1.29, m		1.29, m	0.87, t	1.29, m	1.29, m	1.29, m	0.90, t
19´-CH_3			0.86, d			0.88, d		0.86, d		0.88, d	
20´			1.29, m	1.29, m		0.88, d		1.29, m	1.29, m	0.88, d	
21´			0.87, t	1.29, m				0.87, t	1.29, m		
21´-CH_3				0.86, d					0.86, d		
22´				1.29, m					1.29, m		
23´				0.87, t					0.87, t		

References

1. Hammerschick, T.; Vetter, W. Online hyphenation of centrifugal partition chromatography with countercurrent chromatography (CPC-CCC) and its application to the separation of saturated alkylresorcinols. *Anal Bioanal Chem* 2022, *414*, 5043–5051, doi:10.1007/s00216-022-04136-x.
2. Hammerschick, T.; Wagner, T.; Vetter, W. Isolation of saturated alkylresorcinols from rye grains by countercurrent chromatography. *J Sep Sci* 2021, 1–9, doi:10.1002/jssc.202001230.
3. Englert, M.; Brown, L.; Vetter, W. Heart-cut two-dimensional countercurrent chromatography with a single instrument. *Anal. Chem.* 2015, *87*, 10172–10177, doi:10.1021/acs.analchem.5b02859.
4. Müller, M.; Murić, M.; Glanz, L.; Vetter, W. Improving the resolution of overlapping peaks by heartcut two-dimensional countercurrent chromatography with the same solvent system in both dimensions. *J. Chromatogr. A* 2019, *1596*, 142–151, doi:10.1016/j.chroma.2019.03.012.
5. Friesen, J.B.; Pauli, G.F. Rational development of solvent system families in counter-current chromatography. *J. Chromatogr. A* 2007, *1151*, 51–59, doi:10.1016/j.chroma.2007.01.126.
6. Rüttler, F.; Hammerschick, T.; Schlag, S.; Vetter, W. Isolation of lanosterol and dihydrolanosterol from the unsaponifiable matter of lanolin by urea complexation and countercurrent chromatography in heart–cut recycling mode. *J. Chromatogr. B* 2022, 123470, doi:10.1016/j.jchromb.2022.123470.
